# Timing of Growth Hormone Therapy Initiation and Final Adult Height in Idiopathic Short Stature: A Systematic Review

**DOI:** 10.7759/cureus.117198

**Published:** 2026-09-30

**Authors:** Israa W Khalid, Reem Abbas, Nabaa W Khalid, Samar S Hassan

**Affiliations:** 1 Paediatric Department, Dubai Health Authority, Dubai, ARE; 2 Medical School, Dubai Health Authority, Dubai, ARE; 3 Paediatric Department, Mediclinic City Hospital, Dubai, ARE

**Keywords:** aromatase inhibitor, bone age, final adult height, growth hormone therapy, idiopathic short stature, prepubertal initiation, pubertal timing, systematic review

## Abstract

Idiopathic short stature (ISS) is a height standard deviation score (HtSDS) below −2 SD for age and sex with no identifiable cause. Recombinant human growth hormone (rhGH) is an approved treatment for ISS; however, whether pubertal timing at treatment initiation affects final adult height (FAH) remains an important clinical question. This systematic review aimed to synthesise evidence on the effect of rhGH therapy on FAH in children with ISS, stratified by timing of treatment initiation, and to appraise the methodological quality of the included studies. A systematic search was performed in PubMed, Scopus, and Google Scholar, with predefined keywords and corresponding MeSH terms, including ISS, growth hormone, rhGH, adult height, and timing of therapy. Filters were applied for human studies, paediatric population (<18 years), and publication within the last 10 years. Two reviewers performed study selection and data extraction, and disagreements were resolved through discussion and consensus. In total, 14 studies comprising 5,878 participants across ISS-focused and mixed short-stature cohorts, published between 2016 and 2025 and representing six countries, were included: four randomised controlled trials (RCTs) and 10 observational, cohort, or registry studies. Prepubertal initiation was associated with the most consistent short-term growth responses, although the reported measures differed across studies. The largest registry analysis included 3,939 prepubertal children across diagnostic groups and showed no significant mean gain in near-adult HtSDS in the ISS subgroup. In pubertal boys, combined rhGH and an aromatase inhibitor was associated with an FAH SDS of −1.0 in one randomised trial, while late-pubertal retrospective cohorts reported FAH values of 170.9-173.5 cm in males. One RCT had a low risk of bias, while the remaining RCTs had some concerns, and three observational studies had a serious risk of bias. Earlier rhGH initiation, particularly during the prepubertal period, was associated with more consistent short-term growth responses; however, evidence that earlier initiation leads to superior FAH remains limited and heterogeneous. Further well-designed prospective studies with FAH as a primary endpoint and clearly defined pubertal timing are needed to clarify the effect of treatment timing.

## Introduction and background

Idiopathic short stature (ISS) represents one of the most common reasons for referral to paediatric endocrinology, affecting approximately 1.2-2.5% of children worldwide [1,2]. It is defined by a height standard deviation score (HtSDS) of ≤−2.0 [1], although the U.S. Food and Drug Administration (FDA) uses a threshold of ≤−2.25 for treatment eligibility [3-5], reflecting differences between diagnostic and regulatory criteria. Genetic evaluation is recommended to exclude recognised genetic and syndromic causes of short stature, including chromosomal abnormalities and pathogenic variants associated with growth disorders, before diagnosing ISS [6]. Children diagnosed with ISS generally demonstrate normal growth hormone (GH) secretion under pharmacological stimulation, preserved thyroid and insulin-like growth factor-axis function, and proportionate body habitus; however, they fail to achieve the expected final adult height (FAH) compared to their peers [2,7].

The history and evolution of GH therapy have been well documented [8], and the introduction of recombinant human growth hormone (rhGH) therapy has transformed the management of ISS. Following U.S. FDA approval in 2003, rhGH became a standard therapeutic option for children with ISS and HtSDS ≤−2.25, predicted adult height below −2.25 SDS, and an expectation of not attaining normal adult height [3,4,9]. Multiple randomised trials and meta-analyses have demonstrated that rhGH accelerates height velocity and improves HtSDS during active treatment [5,10,11], with a Cochrane review that included both controlled and uncontrolled studies reporting a mean gain of approximately 3.7 cm in adult height compared with untreated controls [5]. However, the variability in FAH response and the factors determining optimal treatment outcomes remain areas of ongoing investigation [12,13].

Among potential determinants of FAH outcome, the timing of rhGH initiation relative to pubertal development has emerged as a clinically relevant but insufficiently explored factor [14-16]. The pubertal growth spurt represents a critical biological window during which GH-stimulated linear growth potential is greatest and bone maturation accelerates [17,18]. Initiation of therapy during the prepubertal period may maximise cumulative height gain by allowing prolonged exposure to GH stimulation before epiphyseal closure [14]. Conversely, initiation after advanced pubertal progression may limit the remaining growth window and the magnitude of height gain achievable with rhGH therapy [14-16].

Despite this theoretical basis, comparative evidence across pubertal timing subgroups remains fragmented. Existing systematic reviews have largely reported aggregate outcomes without explicit stratification by pubertal timing [5,10], and clinical practice is consequently heterogeneous: some centres initiate rhGH as early as age four to five years, while others defer therapy until height deficit severity justifies the cost and burden of treatment [4,16]. Combination strategies, pairing rhGH with aromatase inhibitors (AIs) to delay epiphyseal fusion [19,20] or GnRH analogues (GnRHa) to slow pubertal tempo [21], have been explored to improve outcomes among patients presenting with advanced skeletal maturation. However, comparative evidence between early rhGH monotherapy and later combination approaches remains limited.

Although rhGH is known to improve growth velocity in children with ISS, it remains uncertain whether treatment initiation at different stages of pubertal development results in meaningful differences in FAH. This uncertainty represents the key evidence gap addressed by the present review.

For the purposes of this review, ISS was defined according to the diagnostic criteria used in the included studies, generally encompassing HtSDS thresholds ranging from ≤−2.0 to ≤−2.25, together with normal GH secretion and the absence of an identifiable pathological cause.

Therefore, this systematic review was designed to address this evidence gap by evaluating FAH outcomes according to the timing of rhGH initiation, incorporating recent registry and real-world data. It also assesses the methodological quality and risk of bias across included studies.

This systematic review aimed to identify and synthesise evidence on the effect of rhGH therapy on FAH and related growth outcomes in children with ISS, with particular emphasis on differences according to the timing of treatment initiation. The review also evaluated methodological quality and risk of bias across the included studies.

## Review

Methodology

This systematic review was conducted and reported in accordance with the Preferred Reporting Items for Systematic Reviews and Meta-Analyses (PRISMA) 2020 statement [22]. The review question and eligibility framework were structured using the PICOT framework (Population, Intervention, Comparator, Outcomes, and Timing), with Study Design considered as an additional eligibility domain, as summarised in Table 1.

**Table 1:** PICOT framework for systematic review. AI = aromatase inhibitor; FAH = final adult height; GH = growth hormone; GnRHa = GnRH analogue; HtSDS = height standard deviation score; HV = height velocity; ISS = idiopathic short stature; NR = not reported; PAH = predicted adult height; RCT = randomised controlled trial; non-RCT = non-randomised controlled trial; ΔHtSDS = change in HtSDS from baseline

Component	Description
Population (P)	Children and adolescents (<18 years) with a diagnosis of ISS, defined by an HtSDS ≤−2.0 to −2.25, normal GH secretion, and absence of an identifiable pathological cause
Intervention (I)	rhGH therapy, administered either as monotherapy or in combination with adjuvant therapies, including AI or GnRHa
Comparator (C)	Placebo, untreated controls, delayed treatment, alternative dosing strategies, alternative formulations, combination regimens, or single-arm pre-post (before-after) comparisons without a concurrent control group
Outcomes (O)	The primary outcome was FAH, defined as study-reported adult height after completion of growth or documented epiphyseal closure. Near-adult height was considered separately when HV was <2 cm/year but epiphyseal closure was not confirmed. PAH was treated as a distinct surrogate outcome. Secondary outcomes included HV, HtSDS, and ΔHtSDS
Timing (T)	Eligible studies were required to define treatment initiation using at least one of the following: chronological age, Tanner stage, or bone age. Treatment timing categories were predefined as prepubertal, pubertal, and late-pubertal
Study design (S)	RCTs, non-RCTs, prospective/retrospective cohorts, registry studies. Excluded: case series (<10 participants), conference abstracts, animal studies

Eligibility Criteria

Population: Eligible participants were children and adolescents under 18 years of age diagnosed with ISS according to published consensus criteria, with HtSDS ≤−2.25 (or ≤−2.0 in studies using different diagnostic thresholds), normal GH secretion, and no identifiable pathological aetiology [1,4]. Diagnostic practices for confirming ISS and excluding alternative causes of short stature were guided by established consensus recommendations [16,23]. ISS-only studies and studies reporting separately extractable ISS-specific outcomes were considered the primary evidence for efficacy. Mixed ISS/growth hormone deficiency (GHD) or mixed short-stature cohorts without separately extractable ISS-specific outcomes were retained only as supportive or contextual evidence and were not included in the ISS-specific efficacy synthesis.

Intervention: Eligible interventions included rhGH therapy, as monotherapy or in combination with an AI (e.g., anastrozole, letrozole) or GnRHa (e.g., leuprolide, triptorelin), regardless of approved dose and duration. Studies were excluded when they reported only non-ISS populations, including Turner syndrome, Prader-Willi syndrome, chronic renal insufficiency, or small for gestational age without extractable ISS-specific outcomes.

Comparator: Any comparator could be used, including placebo, no treatment, delayed treatment, or an active comparator such as an alternative formulation, dose regimen, or combination strategy.

Outcomes: The primary outcome was FAH, defined as study-reported adult height after completion of growth or documented epiphyseal closure [14]. Near-adult height was considered separately when height velocity (HV) was <2 cm/year but epiphyseal closure was not confirmed. Predicted adult height (PAH) was analysed as a distinct surrogate outcome and was not considered equivalent to confirmed FAH. Secondary outcomes comprised change in height standard deviation score (ΔHtSDS) from baseline to the last observation or FAH, HV (cm/year), and attained HtSDS at follow-up. Studies were required to specify treatment initiation using chronological age, Tanner stage, or bone age.

Study Design

Randomised controlled trials (RCTs), non-RCTs, prospective and retrospective cohort studies, and registry studies were eligible. Case series including fewer than 10 participants, conference abstracts without full-text peer-reviewed data, and in vitro or animal studies were excluded.

Search Strategy

Searches were performed in PubMed/MEDLINE, Scopus, and Google Scholar using controlled vocabulary and free-text terms related to ISS, rhGH, adult or final height, and treatment timing. A 10-year publication window was prespecified to focus the review on contemporary clinical evidence, including current rhGH treatment practices, dosing approaches, and adjunctive strategies. Human and paediatric studies were considered according to the predefined eligibility criteria. Google Scholar was used as a supplementary search source rather than as a primary bibliographic database. The first 100 records ranked by Google Scholar relevance were screened using the predefined search terms because of the very large and dynamically ranked result set. Potentially eligible records were incorporated into the screening dataset, and duplicates were identified during the deduplication process in Rayyan. Because Google Scholar result estimates are dynamic and the total displayed hit count was not prospectively recorded, this value could not be reported reliably. In total, 932 records were identified across PubMed/MEDLINE, Google Scholar, and Scopus, comprising 497 from PubMed/MEDLINE, 100 from Google Scholar, and 335 from Scopus. After 79 duplicate records were removed, 853 records remained for screening. The complete PubMed/MEDLINE search terms and database summary are provided in the Appendices.

Study Selection

Two reviewers independently screened titles and abstracts using Rayyan AI software. Following removal of 79 duplicates across all databases, 853 records were screened, and 770 were excluded at title and abstract screening, leaving 83 reports sought for retrieval. All 83 reports were retrieved and underwent full-text eligibility assessment. In total, 69 reports were excluded because they lacked ISS-specific timing data (n = 27), lacked an FAH or HV endpoint (n = 22), involved a non-ISS population (n = 15), or used an ineligible design (n = 5). Therefore, 14 studies met the inclusion criteria. Any disagreements between reviewers were resolved through discussion until consensus was reached.

Data Extraction

Two reviewers independently performed data extraction using a predefined standardised extraction framework. Extracted data included study identification and citation, country and setting, study design, ISS diagnostic criteria, sample size (total, GH-treated, and control groups), intervention and comparator, timing of rhGH initiation (including definition and measurement of time), dosage of rhGH, duration of therapy, follow-up duration and completion rate, primary and secondary outcome measures and results, and statistical methods. Data accuracy was ensured through independent verification, with discrepancies reviewed and resolved between reviewers.

Risk of Bias Assessment

Study-level risk of bias was independently evaluated by the two reviewers, using the revised Cochrane Risk of Bias 2 tool (RoB 2) for RCTs [24] and the Risk of Bias in Non-randomised Studies of Interventions (ROBINS-I) tool for observational studies [25].

For RCTs, RoB 2 evaluated bias arising from the randomisation process, deviations from intended interventions, missing outcome data, measurement of the outcome, and selection of the reported result. For observational studies, ROBINS-I evaluated bias due to confounding, participant selection, intervention classification, deviations from intended interventions, missing data, outcome measurement, and selection of the reported result. Judgments were assigned as low risk, some concerns/moderate risk, or serious risk according to the respective assessment frameworks. Disagreements in risk-of-bias judgments were resolved through discussion and consensus.

Timing-Group Classification

Studies were classified a priori according to the reported timing of rhGH initiation. Tanner pubertal staging was used as the primary classification criterion, where available, based on Tanner’s original five-stage sexual maturity classification [18]. When Tanner stage was not reported, bone age was used as the secondary criterion, informed by the Greulich and Pyle skeletal development atlas [26]. Chronological age was used only when neither Tanner stage nor bone age was available. Prepubertal initiation was defined primarily as Tanner stage I; pubertal initiation as Tanner stages II-IV; and late-pubertal initiation as Tanner stage V. When Tanner stage was unavailable, corresponding bone age criteria were applied, followed by chronological age only as a last-resort classification criterion. Studies spanning more than one predefined timing category, or not reporting outcomes sufficiently by timing to permit a single category assignment, were classified as “Mixed” and described narratively. Where timing indicators were discordant, Tanner stage took precedence over bone age, and bone age took precedence over chronological age.

Synthesis

Due to substantial clinical and methodological heterogeneity across studies, including differences in timing definitions, treatment protocols, comparator groups, follow-up duration, and study designs, a formal meta-analysis was not considered appropriate. A narrative synthesis was therefore performed in accordance with the Synthesis Without Meta-Analysis (SWiM) reporting guideline [27]. Outcomes were synthesised separately according to outcome type, with FAH and FAH SDS considered the primary endpoints, HV considered a secondary endpoint, and HtSDS, ΔHtSDS, PAH, and target height attainment reported as additional outcomes. Measures expressed in different units were not directly compared. Findings were then described according to the timing of rhGH initiation. Formal assessment of publication bias and quantitative sensitivity analyses were not performed because no pooled meta-analysis was undertaken and the included studies were clinically and methodologically heterogeneous.

Results

Study Selection

The primary database search identified 932 records from PubMed (n = 497), Google Scholar (n = 100), and Scopus (n = 335). After removal of 79 duplicate records, 853 records underwent title and abstract screening using Rayyan AI. Of these, 770 records were excluded, leaving 83 reports sought for retrieval. All 83 reports were retrieved and assessed for eligibility. In total, 69 reports were excluded because they lacked ISS-specific timing data (n = 27), lacked an FAH or HV endpoint (n = 22), involved a non-ISS population (n = 15), or used an ineligible design (n = 5). In total, 14 studies were included in the systematic review. The study selection process is presented in Figure 1.

**Figure 1:**
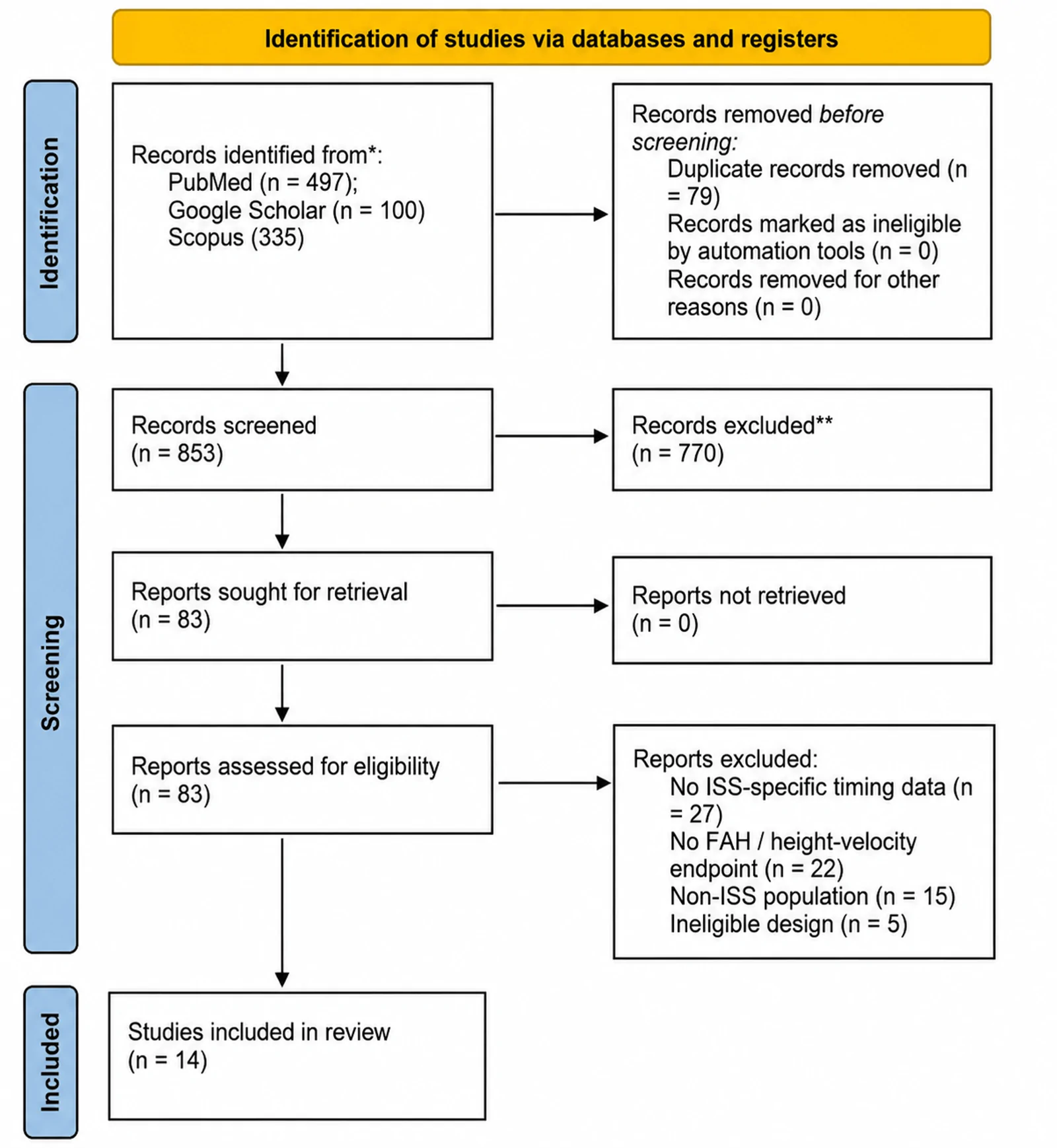
Preferred Reporting Items for Systematic Reviews and Meta-Analyses (PRISMA) 2020 flow diagram of study selection. The primary database search across PubMed, Google Scholar, and Scopus identified 932 records. After removal of 79 duplicates, 853 records were screened; 770 were excluded at title and abstract screening, 83 reports were sought and retrieved, and 69 reports were excluded at full-text assessment. Fourteen studies were included in the final review. Source: Page et al. [22]. This work is licensed under CC BY 4.0. FAH = final adult height; ISS = idiopathic short stature

Characteristics of Included Studies

The 14 included studies were published between 2016 and 2025 and comprised an aggregate of 5,878 participants across ISS-focused and mixed short-stature cohorts. Studies were conducted in China (n = 7), Korea (n = 3), the United States/Chile (n = 1), the Netherlands (n = 1), the United Arab Emirates (n = 1), and multinational settings (n = 1).

Study designs included four RCTs, i.e., Mauras et al. [28], Yuan et al. [29], Chung et al. [30], and Hwang et al. [31], and 10 observational, cohort, or registry-based studies. The observational and registry evidence included Chen et al. [32] and Tamaro et al. [33]. As contextual, non-included background, Wu et al. (2025) [34] analysed real-world rhGH treatment patterns using two Chinese registries (CGSD and CGLS), including 41,942 treated children, of whom 24,445 had ISS; the study evaluated treatment initiation patterns, rhGH formulation, dosing, and adherence but included mixed short-stature aetiologies rather than an ISS-only population. Additional included studies were Wang et al. [35] and Li et al. [36]. Related formulation evidence from Bin-Abbas and Jabari [37] was used for contextual comparison. The remaining included studies comprised Han et al. [38], Yoon et al. [39], Kruijsen et al. [40], Al Jneibi et al. [41], and Cui et al. [42]; Chae et al. [43] provided additional contextual evidence, and Gou et al. [44] was included among the real-world cohorts. Sample sizes ranged from 42 to 3,939 participants. Treatment duration varied from 26 weeks to approximately nine years. FAH outcomes were reported in five studies, while target height was achieved in one study. Several studies evaluated intermediate outcomes, including HV and HtSDS. Detailed study characteristics are presented in Table 2.

**Table 2:** Characteristics of included studies. “Mixed short-stature cohort including ISS and GHD” indicates that the reported study population was not restricted to ISS. “Mixed” under timing group indicates that participants spanned more than one predefined pubertal timing category and the reported outcomes were not stratified sufficiently to assign the study to a single timing group. AI = aromatase inhibitor; BA = bone age; FAH = final adult height; GH = growth hormone; GHD = growth hormone deficiency; GnRHa = GnRH analogue; HtSDS = height standard deviation score; HV = height velocity; ISS = idiopathic short stature; KIGS = Pfizer International Growth Study; NR = not reported; RCT = randomised controlled trial; SDS = standard deviation score

Study ID	Study design	Country	Population (N)	Age at start	Timing group	Intervention	Primary growth outcome
Mauras et al., 2016 [28]	RCT, three-arm	USA/Chile	ISS boys (n = 76)	14.1 ± 0.1 years	Pubertal (Tanner 2–4)	GH vs. AI vs. GH + AI	FAH SDS −1.0 (GH + AI)
Yuan et al., 2022 [29]	Phase 3 RCT	China	ISS (n = 481)	~5–9 years	Prepubertal	GH vs. placebo	ΔHtSDS +1.0 (SD 0.31)
Chung et al., 2018 [30]	RCT	Korea	ISS (n = 90)	~6–7 years	Prepubertal (Tanner I)	GH vs. delayed GH	HV +3.47 cm/year
Hwang et al., 2018 [31]	RCT	Korea	ISS (n = 46)	~5–6 years	Prepubertal (Tanner I)	Weekly vs. daily GH	HV 3.6–5.1 cm/year
Wang et al., 2023 [35]	Retrospective cohort	China	ISS males, adv. BA (n = 68)	BA 13–15 years	Late pubertal (BA ≥13)	GH ± AI ± GnRHa	FAH 170.9–173.5 cm
Chen et al., 2024 [32]	Retrospective, before-after	China	ISS (n = 42)	8.93±1.52 years	Prepubertal	GH monotherapy	HtSDS −2.41 → −1.33
Li et al., 2024 [36]	Retrospective cohort	China	ISS + GHD (n = 90)	~8.5 years	Unclassifiable	GH monotherapy	Improved HV (NR)
Gou et al., 2022 [44]	Real-world cohort	China	ISS + GHD (n = 174)	~5.6 years	Unclassifiable	GH monotherapy	Improved HtSDS (NR)
Han et al., 2022 [38]	Retrospective cohort	Korea	ISS (n = 95)	~10 years	Prepubertal	GH vs. routine care	HV 8.53 vs. 6.09 cm/year
Yoon et al., 2022 [39]	Registry cohort	Korea	ISS (n = 182)	7.29 ± 2.60 years	Prepubertal	GH monotherapy	Improved HtSDS (NR)
Tamaro et al., 2025 [33]	Retrospective KIGS registry analysis	Multinational	KIGS mixed diagnostic cohort (n = 3,939; ISS n = 1,238)	~8–9 years	Prepubertal	GH monotherapy	No significant near-adult HtSDS gain in the ISS subgroup
Kruijsen et al., 2025 [40]	Prospective cohort	Netherlands	ISS (n = 129)	8.0 ± 2.8 years	Mixed	GH monotherapy	FAH gain +2.1 SDS
Cui et al., 2025 [42]	Retrospective	China	ISS adolescents (n = 96)	~13 years	Late pubertal (BA ≥13)	GH + AI/GnRHa	FAH ~173.2 cm
Al Jneibi et al., 2025 [41]	Registry cohort	UAE	Mixed short-stature cohort including ISS and GHD (n = 370)	~11.1 years	Mixed	GH monotherapy	>90% reached target height

Risk of Bias

Risk-of-bias assessments are summarised in Table 3 and presented separately by study design in Figure 2 and Figure 3. Among the four RCTs, Yuan et al. (2022) [29] was judged to have a low risk of bias across all RoB 2 domains. Mauras et al. (2016) [28], Chung et al. (2018) [30], and Hwang et al. (2018) [31] were assessed as having some concerns, primarily because of limitations in reporting or blinding and, for some studies, limited follow-up for FAH assessment.

**Table 3:** Risk of bias summary. FAH = final adult height; GHD = growth hormone deficiency; ISS = idiopathic short stature; RCT = randomised controlled trial; RoB 2 = Risk of Bias 2 tool; ROBINS-I = Risk of Bias in Non-randomised Studies of Interventions

Study	Design	RoB tool	Judgment	Key concern(s)
Mauras et al., 2016 [28]	RCT	RoB 2	Some concerns	Three-arm design; allocation concealment and participant blinding not fully clear
Yuan et al., 2022 [29]	RCT	RoB 2	Low risk	Large multicentre, placebo-controlled RCT; robust randomisation; low attrition
Chung et al., 2018 [30]	RCT	RoB 2	Some concerns	Small sample; 12-month duration insufficient for FAH; limited blinding detail
Hwang et al., 2018 [31]	RCT	RoB 2	Some concerns	Dose/formulation comparison; limited blinding; no FAH endpoint
Wang et al., 2023 [35]	Retrospective cohort	ROBINS-I	Serious risk	Non-randomised; advanced bone age selection bias; residual confounding
Chen et al., 2024 [32]	Before-after study	ROBINS-I	Serious risk	No concurrent control group; pre-post design; high confounding potential
Li et al., 2024 [36]	Retrospective cohort	ROBINS-I	Serious risk	Mixed ISS + GHD population; ISS subgroup data not separately extractable
Gou et al., 2022 [44]	Real-world cohort	ROBINS-I	Moderate risk	Observational; ISS vs. GHD comparison; some confounders controlled
Han et al., 2022 [38]	Retrospective cohort	ROBINS-I	Moderate–serious risk	Non-randomised control group; residual confounding; short follow-up
Yoon et al., 2022 [39]	Registry cohort	ROBINS-I	Moderate risk	Large nationwide registry; selection bias likely; no control group
Tamaro et al. 2025 [33]	Registry (KIGS)	ROBINS-I	Moderate risk	Very large dataset; heterogeneous protocols; confounding by indication
Kruijsen et al., 2025 [40]	Prospective cohort	ROBINS-I	Moderate risk	Well-designed cohort; confounding by indication; no randomisation
Cui et al., 2025 [42]	Retrospective	ROBINS-I	Moderate risk	Combination therapy cohort; selection bias in late-puberty cases
Al Jneibi et al. 2025 [41]	Registry cohort	ROBINS-I	Moderate risk	Multicentre registry; protocol variability across centres

**Figure 2:**
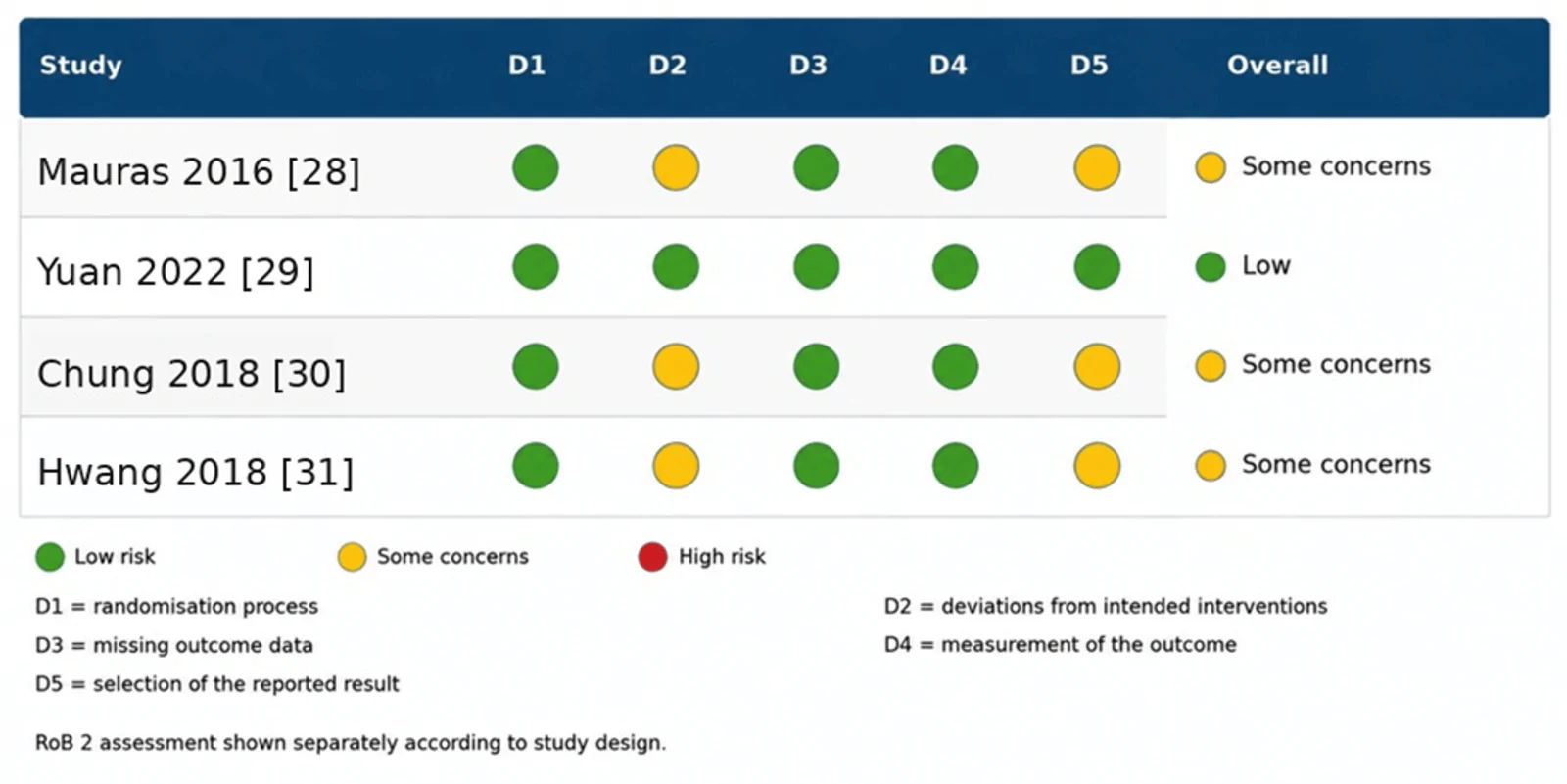
Risk of Bias 2 (RoB 2) risk-of-bias assessment for randomised controlled trials. D1 = randomisation process; D2 = deviations from intended interventions; D3 = missing outcome data; D4 = measurement of the outcome; D5 = selection of the reported result. Green = low risk; yellow = some concerns; red = high risk. Study reference numbers are shown in square brackets.

**Figure 3:**
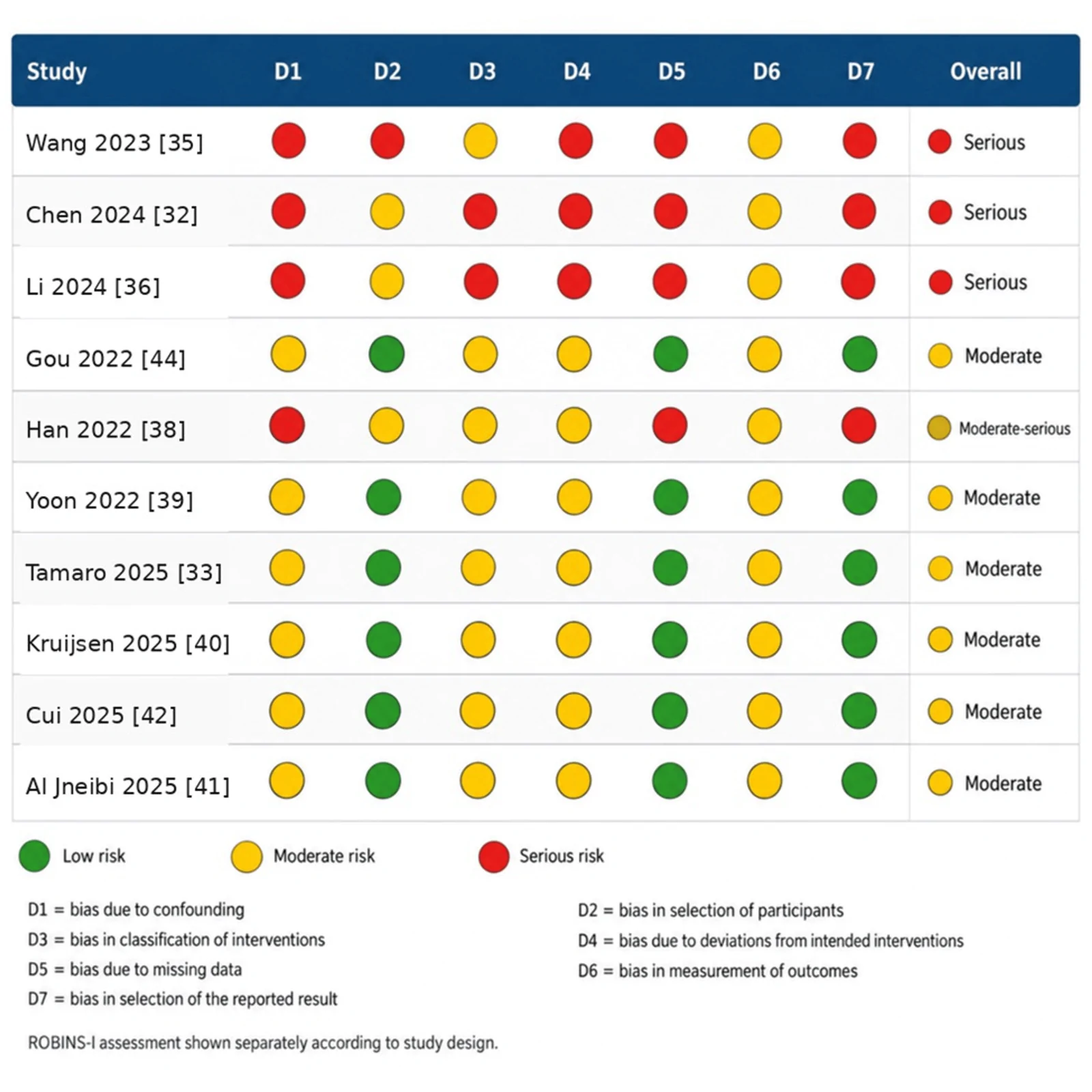
Risk of Bias in Non-randomised Studies of Interventions (ROBINS-I) risk-of-bias assessment for observational and registry studies. D1 = confounding; D2 = selection of participants; D3 = classification of interventions; D4 = deviations from intended interventions; D5 = missing data; D6 = measurement of outcomes; D7 = selection of the reported result. Green = low risk; yellow = moderate risk; red = serious risk. Study reference numbers are shown in square brackets.

Among the observational studies, three were judged to have a serious risk of bias [32,35,36]. Major concerns included the absence of a concurrent control group in a before-and-after study design [32], non-randomised treatment allocation with potential selection bias related to advanced bone age [35], and inclusion of a mixed ISS and GHD population without separately extractable ISS-specific outcomes [36]. No observational study was considered to have a low risk of bias according to ROBINS-I criteria.

Outcomes by Timing Subgroup

Prepubertal initiation: Seven studies were classified as prepubertal: Yuan et al. (2022) [29], Chung et al. (2018) [30], Hwang et al. (2018) [31], Chen et al. (2024) [32], Han et al. (2022) [38], Yoon et al. (2022) [39], and Tamaro et al. (2025) [33]. These studies predominantly evaluated rhGH therapy initiated during the prepubertal period, with mean baseline ages ranging from approximately 5.6 to 10 years. The largest RCT in this subgroup was a multicentre, Phase III, placebo-controlled trial involving 481 children with ISS [29]. After 52 weeks of treatment, the rhGH group demonstrated a mean increase in HtSDS of +1.0 (SD = 0.31).

Chung et al. (2018) [30] randomised 90 prepubertal children with ISS to immediate or delayed rhGH treatment, with the immediate-treatment group showing an HV of 3.47 cm/year greater than the delayed-treatment group. Hwang et al. (2018) [31] reported HV values of approximately 3.6-5.1 cm/year with once-weekly sustained-release and daily rhGH regimens.

Among observational studies, Chen et al. (2024) [32] reported an improvement in HtSDS from −2.41 to −1.33 following treatment, while Han et al. (2022) [38] reported HVs of 8.53 cm/year in rhGH-treated children compared with 6.09 cm/year in the control group. Yoon et al. (2022) [39] reported increasing HtSDS during follow-up, whereas Tamaro et al. (2025) [33] reported no significant mean gain in near-adult HtSDS in the ISS subgroup.

Pubertal initiation: Mauras et al. (2016) [28] conducted the only RCT in this review specifically evaluating rhGH treatment initiated during puberty and reporting FAH as a primary outcome. The study included 76 male adolescents with ISS (mean age = 14.1 ± 0.1 years; Tanner stages 2-4) randomised to rhGH monotherapy, AI monotherapy, or combined rhGH plus AI therapy. The combination therapy group achieved a mean FAH SDS of −1.0, representing the most favourable outcome among the treatment groups.

Kruijsen et al. (2025) [40] and Al Jneibi et al. (2025) [41] were not treated as direct evidence for a single pubertal-initiation category. Both cohorts included participants spanning more than one pubertal stage, and timing-specific final height outcomes were not sufficiently separable for a single-category assignment. They are therefore classified as Mixed and summarised in the Mixed and Unclassifiable Timing section.

Late-pubertal initiation: Two retrospective cohort studies evaluated treatment initiation in adolescents with advanced skeletal maturation [35,42]. Wang et al. (2023) [35] analysed 68 male adolescents with ISS and bone ages between 13 and 15 years who received rhGH alone or in combination with AI and/or GnRHa therapy. Mean FAH ranged from 170.9 cm in the rhGH monotherapy group to 173.5 cm in the group receiving combined rhGH, AI, and GnRHa therapy.

Similarly, Cui et al. (2025) [42] reported a mean FAH of approximately 173.2 cm in 96 male adolescents with ISS and bone age ≥13 years treated with rhGH-based regimens that included an AI and, in one group, a GnRHa.

Mixed and unclassifiable timing: Four studies were not readily assignable to a single timing subgroup because participants were recruited across a broad age range or timing-specific outcomes were not reported. Kruijsen et al. (2025) [40] reported a mean FAH gain of +2.1 SDS following long-term growth hormone therapy, while Al Jneibi et al. (2025) [41] found that more than 90% of participants achieved their calculated target height range. Li et al. (2024) [36] was retained only as supportive contextual evidence because the study included a mixed ISS/GHD population and ISS-specific outcomes were not separately extractable. Gou et al. (2022) [44] also provided supportive evidence, although timing-specific analyses were unavailable.

Synthesis Approach

Substantial clinical and methodological heterogeneity was identified across the included studies, including differences in study design, participant characteristics, rhGH dosing regimens, treatment duration, timing definitions, comparator groups, and outcome measures. Consequently, quantitative meta-analysis was not considered appropriate.

A narrative synthesis was performed in accordance with the SWiM reporting guideline [27]. Outcomes were synthesised separately according to outcome type, with FAH and FAH SDS considered the primary endpoints, HV considered a secondary endpoint, and HtSDS, ΔHtSDS, PAH, and target height attainment reported as additional outcomes. Measures expressed in different units were not directly compared. Findings were then described according to the timing of rhGH initiation.

Comparative Final Adult Height Outcomes by Timing Subgroup

Direct statistical comparison between timing groups was not possible because of heterogeneity in study populations, treatment protocols, outcome definitions, and reporting methods. Earlier initiation was generally associated with larger short-term growth responses, but whether this translates into superior FAH remains uncertain.

Across studies, the most extensive evidence base was available for prepubertal treatment initiation, with generally favourable short-term growth outcomes. Direct pubertal initiation evidence for FAH was limited primarily to the Mauras et al. randomised trial. Kruijsen et al. and Al Jneibi et al. were retained as mixed-timing evidence because their cohorts crossed timing categories and did not provide sufficiently separable timing-specific final height estimates (Table 4).

**Table 4:** Summary of FAH, near-adult height, and other growth outcomes by timing subgroup. AI = aromatase inhibitor; BA = bone age; FAH = final adult height; GnRHa = GnRH analogue; HtSDS = height standard deviation score; HV = height velocity; NR = not reported; RCT = randomised controlled trial; SDS = standard

Timing group	Key studies	Reported growth outcome	Key observations
Prepubertal (Tanner I; age <8–9 years)	Yuan et al., 2022 [29]; Chung et al., 2018 [30]; Hwang et al., 2018 [31]; Chen et al., 2024 [32]; Han et al., 2022 [38]; Yoon et al., 2022 [39]; Tamaro et al., 2025 [33]	Improvements in HtSDS and HV were reported across studies. Hwang et al. reported HV values of approximately 3.6–5.1 cm/year, and Han et al. reported 8.53 cm/year in rhGH-treated children. Tamaro et al. reported no significant mean near-adult HtSDS gain in the ISS subgroup	Consistent improvements in short-term growth parameters were reported. Most prepubertal studies evaluated rhGH monotherapy. Large-sample near-adult height data were available from the KIGS registry
Pubertal (Tanner II–IV; BA ~9–13 years)	Mauras et al., 2016 [28]	FAH SDS −1.0 (GH + AI, Mauras et al.)	Direct pubertal initiation FAH evidence was limited to the Mauras et al. RCT. Kruijsen et al., 2025 [40] and Al Jneibi et al., 2025 [41] are classified as Mixed and are not used as direct pubertal evidence
Mixed timing	Kruijsen et al., 2025 [40]; Al Jneibi et al., 2025 [41]	FAH gain +2.1 SDS (Kruijsen et al.); >90% achieved calculated target-height range (Al Jneibi et al.)	These cohorts crossed more than one timing category and did not provide sufficiently separable timing-specific final height estimates. They are contextual mixed-timing evidence, not direct pubertal evidence
Late-pubertal (Tanner V / BA ≥13 yr)	Wang et al., 2023 [35]; Cui et al., 2025 [42]	FAH 170.9–173.5 cm in males (Wang et al., 2023); FAH ~173.2 cm (Cui et al., 2025)	Near-normal adult height was reported in selected adolescents with advanced skeletal maturation. Evidence is limited to retrospective studies with moderate or serious risk of bias. Combination regimens were used in the available studies, but causal benefit cannot be inferred from these cohorts
Unclassifiable timing subgroup	Li et al., 2024 [36]; Gou et al., 2022 [44]	Improved HtSDS and HV (exact values NR)	Timing definitions were insufficient for subgroup allocation. These studies provide supportive or contextual evidence only and were not used for timing-specific efficacy conclusions

Discussion

Principal Findings

This systematic review synthesised evidence from 14 studies with an aggregate of 5,878 participants across ISS-focused and mixed short-stature cohorts treated with rhGH, with outcomes specifically stratified according to timing of treatment initiation across three predefined subgroups.

The available evidence suggests that earlier initiation of rhGH therapy, particularly during the prepubertal period, is associated with larger short-term improvements in HV. Evidence for a corresponding advantage in FAH is less certain because few studies directly compared timing groups and the available FAH data were heterogeneous. The biological rationale for earlier treatment remains plausible, as a longer period of GH-mediated linear growth is available before epiphyseal maturation and closure [14], but the present evidence does not establish a causal timing effect.

Studies of prepubertal initiation consistently reported improvement in short-term growth measures, although the specific outcomes and units differed across studies [29-31]. Yuan et al. reported change in HtSDS, whereas Chung et al. and Hwang et al. primarily reported HV outcomes; these measures were therefore interpreted separately rather than combined as a single effect range. The largest available near-adult-height dataset, derived from the Tamaro et al. (2025) KIGS analysis (n = 3,939 across three diagnostic groups), found no statistically significant mean gain in height SDS at near-adult height in the ISS subgroup [33]. A separate Korean multicentre cohort likewise found that children who began GH treatment before six years of age attained higher HtSDS than those who started later, supporting the importance of earlier treatment initiation [43].

Among pubertal initiators, the highest-quality FAH-focused randomised trial (Mauras et al., 2016) demonstrated that combining rhGH with an AI resulted in an FAH SDS of −1.0 [28].

Late-pubertal cohorts receiving rhGH with adjunctive regimens reported mean FAH values of 170.9-173.5 cm in males (Wang et al., 2023; Cui et al., 2025) [35,42]. These findings indicate that additional growth can occur despite advanced skeletal maturation, but they should not be interpreted as evidence that late combination treatment causes a clinically meaningful FAH gain. Both studies were retrospective, treatment allocation was non-randomised, and direct comparison with earlier initiation was not available [35,42].

Comparison With Previous Systematic Reviews

Previous systematic reviews evaluating rhGH in ISS have reported average FAH gains of 3.5-7.5 cm over untreated natural history; however, these analyses generally reported pooled outcomes without stratification according to pubertal timing at treatment initiation [5,10].

Network meta-analysis suggests potential benefit, although direct ISS trials remain limited [45]. Deodati and Cianfarani (2011) reported that the average FAH improvement was approximately 4.2 cm following rhGH therapy, although substantial variability was observed across studies [10]. Similarly, the Cochrane review by Bryant et al. (2007) also showed a small but significant improvement in FAH with rhGH compared to placebo [5]. A key limitation of these later reviews was the absence of treatment timing stratification, which restricts their ability to determine whether earlier initiation provides superior adult height outcomes.

The present review extends existing evidence by using the timing of initiation as the primary stratification variable and incorporating recent studies published during 2024-2025, including registry-based and real-world evidence.

Clinical Implications

The findings support careful early evaluation of children with persistent short stature, while treatment decisions should remain individualised and should not be based on timing alone. Pubertal stage, bone age, predicted remaining growth, diagnosis, treatment burden, expected benefit, and patient preferences should be considered together. Pubertal timing at rhGH initiation should be documented consistently in future trials and registries. For adolescents presenting with advanced bone age, retrospective studies of rhGH combined with an AI or GnRH analogue report favourable adult height values [35,42], but the evidence is insufficient to infer a causal benefit or to recommend these regimens routinely based on this review alone.

Limitations

This review has several important limitations. The included studies were heterogeneous in design, ISS diagnostic criteria, definitions of treatment timing, outcome measures, GH doses, and intervention regimens, which precluded a formal meta-analysis. Most included studies were observational or retrospective, and three were judged to have a serious risk of bias using ROBINS-I. Only two studies specified FAH as a primary endpoint with clearly defined pubertal timing at treatment initiation [28,35]. Generalisability is also limited because much of the available evidence originated from East Asian populations, particularly China and Korea. Variability in rhGH dosing protocols may have contributed to differences in treatment response, and certain long-term safety outcomes remain insufficiently evaluated in ISS-specific trials and systematic reviews. This systematic review was not prospectively registered in PROSPERO or another systematic review registry, which represents a limitation of the study. The search was limited to PubMed/MEDLINE, Scopus, and Google Scholar and did not include Embase, Web of Science Core Collection, CENTRAL, ClinicalTrials.gov, or WHO International Clinical Trials Registry Platform. In addition, a prespecified 10-year publication window was applied to focus the review on contemporary clinical evidence. This restriction may have excluded older foundational trials reporting adult height outcomes and therefore limits the comprehensiveness of the evidence base. Older studies were used where relevant to provide clinical and historical context but were not part of the predefined systematic review eligibility window. These limitations should be considered when interpreting the timing-specific conclusions. A formal certainty-of-evidence assessment using the Grading of Recommendations Assessment, Development, and Evaluation framework was not performed, which limits the strength of inference regarding the major treatment timing conclusions. Long-term safety evidence relevant to this context includes broad GH surveillance, an ISS-specific paediatric safety analysis, and mortality follow-up cohorts of childhood GH recipients [46-49]. The original database search dates and the verbatim Google Scholar and Scopus search strings were not prospectively archived, which limits full reproducibility of the search.

Priorities for Future Research

Future research should prioritise adequately powered prospective RCTs that evaluate FAH as a primary endpoint and use prespecified stratification by pubertal timing. ISS trials and registries should apply standardised definitions of treatment timing based on Tanner stage, bone age, and chronological age. International longitudinal registries should use consistent protocols and systematic documentation of pubertal status to permit large-scale timing-stratified FAH analyses. Long-term studies are also needed to evaluate the safety of AI co-administration in adolescent males and to investigate genetic and clinical predictors of GH response and bone age-based prediction tools [50-53]. More geographically and ethnically diverse cohorts are required to improve the external validity and global applicability of the evidence.

## Conclusions

This systematic review provides a time-stratified synthesis of the available evidence regarding the effects of rhGH therapy in children with ISS. Across the included studies, earlier rhGH initiation, particularly during the prepubertal period, was associated with greater short-term growth responses, including improvements in HV and HtSDS. However, evidence that earlier initiation translates into superior FAH remains limited and heterogeneous. Among pubertal patients, favourable adult height outcomes were reported in selected studies. In adolescents with advanced skeletal maturation, combination regimens involving rhGH with an AI or GnRHa were evaluated and may offer additional height benefit. However, the evidence supporting these approaches remains limited and is derived largely from observational or retrospective studies; therefore, the findings should be interpreted cautiously. Overall, the evidence base is limited by methodological heterogeneity, inconsistent reporting of pubertal timing, variation in outcome definitions, and a relative lack of high-quality prospective studies evaluating FAH as a primary endpoint. Future research should prioritise adequately powered prospective studies using standardised definitions of treatment timing and prespecified assessment of FAH. Additional long-term and geographically diverse studies are needed to clarify the effect of treatment timing and the potential role of adjunctive therapies in children and adolescents with ISS.

## Appendices

PubMed/MEDLINE search strategy

Database: PubMed/MEDLINE | Filters: human studies, paediatric population (up to 18 years), publication within the last 10 years.

The PubMed/MEDLINE search combined three concepts. The ISS concept used (“idiopathic short stature”[MeSH Terms] OR “idiopathic short stature”[tiab]); the growth-hormone concept used (“growth hormone”[MeSH Terms] OR “recombinant human growth hormone”[tiab] OR “rhGH”[tiab] OR “growth hormone therapy”[tiab]); and the outcome/timing concept used (“adult height”[tiab] OR “final adult height”[tiab] OR “outcome”[tiab] OR “timing”[tiab] OR “treatment initiation”[tiab]). These three concepts were combined with AND.

The same keyword structure was adapted for Google Scholar and Scopus. The combined database search produced 932 records from PubMed, Google Scholar, and Scopus (497, 100, and 335 records, respectively). Filters were limited to human studies, paediatric population (up to 18 years), and publications within the last 10 years.

**Table 5:** Search strategy summary by database. Full search syntax for PubMed/MEDLINE is provided above. Google Scholar and Scopus searches were adapted using the same core terms and were incorporated into the combined database search. MeSH = Medical Subject Headings; tiab = title/abstract field tag

Database	Syntax summary	Limits
PubMed/MEDLINE	MeSH/keyword terms: idiopathic short stature; growth hormone; recombinant human growth hormone; adult height; outcome; timing	Human studies; paediatric population (up to 18 years); last 10 years; 497 records
Google Scholar	Keyword terms adapted from PubMed strategy	Human studies; paediatric population; last 10 years; 100 records
Scopus	Keyword terms adapted from the core strategy; eligible RCT, cohort, and registry studies considered	Human studies; paediatric population; last 10 years; 335 records
Rayyan AI screening	Title and abstract screening of records remaining after duplicate removal	853 records screened; 83 retained for full-text assessment
